# Shine & Lal index as a predictor for early detection of β-thalassemia carriers in a limited resource area in Bandung, Indonesia

**DOI:** 10.1186/s12881-019-0868-x

**Published:** 2019-08-09

**Authors:** Ani Melani Maskoen, Lelani Reniarti, Edhyana Sahiratmadja, Joice Sisca, Sjarif Hidajat Effendi

**Affiliations:** 1 Department of Oral Biology, Faculty of Dentistry, Universitas Padjadjaran, Bandung, Indonesia; 2 Department of Pediatrics, Dr. Hasan Sadikin General Hospital/Faculty of Medicine, Universitas Padjadjaran, Bandung, Indonesia; 3 Department of Biomedical Sciences, Faculty of Medicine, Universitas Padjadjaran, Bandung, Indonesia; 4 Biotechnology Master Program, Postgraduate School, Universitas Padjadjaran, Bandung, Indonesia

**Keywords:** HbA_2_, HbE, IVS1nt5, β-Thalassemia

## Abstract

**Background:**

Thalassemia is the most common inherited disease in the world, involving α- or β-globin in red blood cells. Thalassemia cases rank fifth in the list of national catastrophic diseases in Indonesia; however, nationwide screening for thalassemia carriers is not yet mandatory. This study aimed to assess whether blood count metrics, such as the Shine & Lal index (SLI; MCV*MCV*MCH/100), might serve as a predictor to screen thalassemia carriers in a limited resource area where molecular methods are not readily available.

**Methods:**

During a family gathering of thalassemia patients, family members (n196) underwent a complete blood count test. Those with MCV < 80 fL and/or MCH < 27 pg and/or SLI < 1530 were further examined for Hb analysis. Only samples with HbA2 fraction > 4% or with a peak in the HbE fraction were sequenced to confirm β-globin gene mutations.

**Results:**

Of 196 family members, 117 (59.6%) had low MCV and/or low MCH and/or low SLI. The HbE fraction (mean 24.06% ± 0.95, range 22.4–26.5) was found in 27 (13.7%) cases, and *all* had a mutation at codon (CD)26 (c.79G > A). The mean HbA_2_ fraction in these samples was 3.18% ± 0.62 (range 2.6–3.8). For samples with HbA2 > 4% (n30; 15.3%), *all* had mutations at IVS1nt5 (c.92 + 5 G > C; n28), CD8/9 (c.27_28insG; n1) and CD19 (c.59A > G; n1). The mean HbA_2_ fraction with a mutation at IVS1nt5 (c.92 + 5 G > C) was 4.65% ± 0.77 (range 4.0–5.6). Interestingly, anaemia was only present in 25 and 57% of β-thalassemia carriers with mutations at CD26 (c.79G > A) and at IVS1nt5 (c.92 + 5 G > C), respectively.

**Conclusions:**

The Shine & Lal index is helpful in the early screening of β-thalassemia carriers, since this index confirms mutations at CD-26 (c.79G > A) and at IVS1nt5 (c.92 + 5 G > C), which are both common mutations in Bandung, Indonesia. Further DNA analysis is a topic of interest to map variants in globin genes and their distribution across populations.

## Background

Thalassemia is an inherited disease caused by mutations in the α- or β-globin gene. The two α- and two β-globin chains form tetramers of globin, and together with haem and iron, they form the haemoglobin (Hb) molecule of red blood cells in normal adults [1]. The α-globin gene cluster is located on chromosome 16 (16p13.3), whereas the β-globin gene cluster is located on chromosome 11 (11p15.5). Mutation or deletion in the α- or β-globin gene may result in a reduction or an absence of globin production, leading to a low Hb concentration. Mutations in the β-globin gene (HBB, NM_000518) are common in populations around the Mediterranean area, the Middle East, Central Asia and the Far East, as well as in Southeast Asia, including Indonesia [2]. Homozygous individuals carrying mutations in the β-globin gene are designated as β-thalassemia major patients, and this patient requires lifelong regular blood transfusion [3]. The heterozygous individual carrying a β-globin gene mutation is known as a thalassemia carrier or trait. This thalassemia carrier passes the mutation to their offspring in an autosomal recessive manner [3]. Since β-thalassemia major cases are increasing, early detection of heterozygous individuals carrying β-globin gene mutations is a point of interest.

In Indonesia, the prevalence of anaemia is high. Anaemia in this region is often described as anaemia due to iron deficiency, especially in children and young women [4]. Although anaemia has been declining over the years, it remains a moderate public health problem. Because Indonesia is located along the ‘thalassemia belt’ area, low Hb may reflect other red blood disorders, such as haemoglobinopathies [2]. However, screening of thalassemia carriers in Indonesia is only sporadically conducted.

A complete blood count test, including Hb, mean corpuscular volume (MCV), and mean corpuscular haemoglobin (MCH), is a routine blood examination in clinical laboratories. Several haematological indices are introduced to categorize microcytic anaemia into an iron deficiency anaemia (IDA) or a carrier state of β-thalassemia, such as the Mentzer index (MCV/RBC), Srivastava index (MCH/RBC), Shine & Lal index (MCV [2]*MCH/100) and many other indices [5]. However, the sensitivity and specificity of these indices vary across regions [6]. Although haematological indices can be used to diagnose presumptive β-thalassemia carriers, a recent development shows that Hb analysis, including fractions of HbA2, HbA, or HbF, may confirm or exclude the β-thalassemia carrier state. Moreover, new technology can detect the form of the HbE fraction [7], thus recommending Hb analysis for further confirmation results [8, 9]. Moreover, because more than 200 different thalassaemic mutations have been reported in the β-globin gene, it is necessary to confirm the diagnosis and to map the mutation spectrum among populations. In a limited resource area such as Indonesia, Hb analysis and molecular examination are expensive. Therefore, this study aimed to explore whether the Shine & Lal index might have a beneficial factor as a predictor to detect a carrier state of thalassemia carriers in the general population, especially in areas where molecular examination is not readily available. Furthermore, common mutations among β-thalassemia carriers were described.

## Methods

### Participants and sample collection

First degree family members, including father and/or mother and/or siblings, were invited to participate in the screening of thalassemia carrier status during a family gathering of thalassemia patients in Dr. Hasan Sadikin General Hospital, Bandung, Indonesia. In this family gathering, a mini seminar was held and involved professional speakers, including a paediatrician, clinical pathologist, and the Indonesian Thalassemia Foundation. Written consent was obtained from all adult participants. A form letter containing a carrier screening agreement for children younger than 16 years old was signed by their parents.

**Fig. 1:**
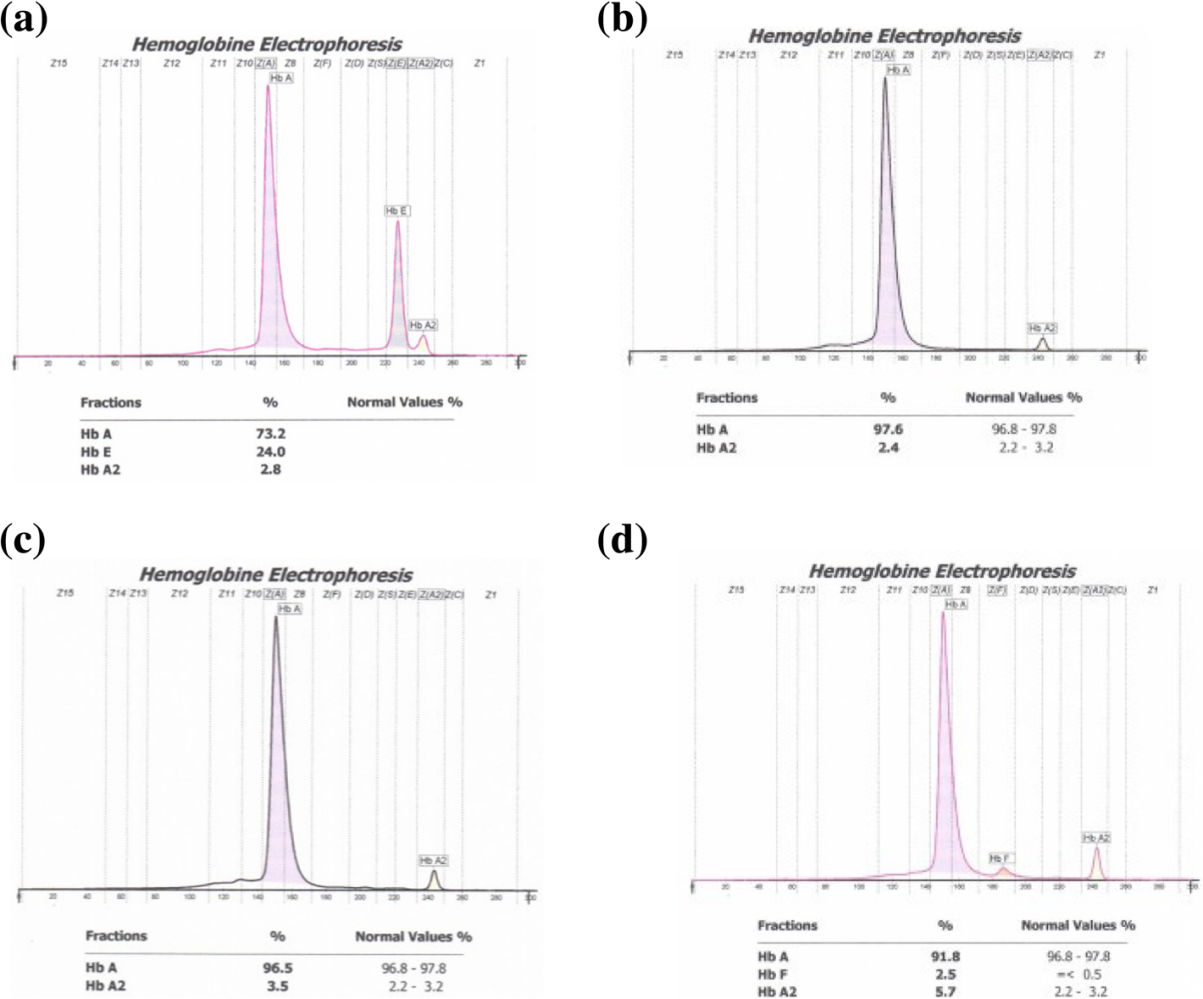
Peaks of various Hb fractions (HbA, HbA2, HbF, and HbE) by capillary electrophoresis. HbE is clearly shown next to HbA2 (**a**), and *all* samples have mutations at c.79G > A (CD26). HbA2 is normal (2.2–3.2%) and borderline (3.2–3.9%), as shown in (**b**) and (**c**), respectively. HbA2 is high (> 4%) (**d**), and most samples have mutations at c92 + 5G > C (IVS1nt5)

Venous blood was collected in a 3 mL EDTA tube, and the complete blood count was examined, including Hb, MCV and MCH. Anaemia was defined according to WHO criteria: Hb < 13 g/dL and < 12 g/dL for males and females, respectively. Only those who had MCV < 80 fL and/or MCH < 27 pg and/or Shine & Lal index < 1530 were further subjected to Hb analysis using capillary electrophoresis (Minicap Sebia, France). This machine could separate the HbE fraction from HbA2; therefore, the sample with the HbE value was further sequenced (Fig. 1a). The other samples were grouped based on HbA2 fraction values of normal (2.2–3.2%), borderline (3.3–3.9%) and high (> 4%) as shown in Fig. 1b, c, and d. Only samples with high HbA2 (> 4%) (Fig. 1d) were further examined for common β-globin gene mutations due to limited financial sources.

The protocol of the study was approved by the Ethics Committee of the Faculty of Medicine, Universitas Padjadjaran, with ethical clearance no. 966/UN6.C.10/PN/2017.

### Detection of mutations in the β-globin gene

DNA was isolated from EDTA venous blood, and the β-globin gene (HBB gene; NM_000518) was amplified using the forward and reverse primer sets as follows: forward primer TLF62028 (ComC) 5’ACCTCACCCTGTGGAGCCAC3’, and reverse primer TLR62320 5’CTATTGGTCTCCTTAAACCTGTCTTGTAACCTTGCTA3’. Polymerase chain reaction was carried out on a thermal cycler (GeneAmp® PCR system 9700, Applied Biosystems) using the following conditions: initial denaturation at 95C for 5 min, followed by 35 cycles at 95C for 30 s, 68C for 30 s, 72C for 1 min, and a final extension at 72C for 5 min. The PCR band was then visualized in 2% agarose gel, running at 50 V for 30 min before further sequencing (First Base, Malaysia). The Hb fractions (HbA2, HbA and HbE) were compared between groups of common β-globin mutations (c.79G > A vs. c92 + 5G > C) as previously described [10].

### Statistical analyses

Anaemia prevalence was compared between groups of common β-globin mutations (c.79G > A vs. c92 + 5G > C) using the χ-square test. The values of the Hb fractions (HbA2, HbA and Hb E) were checked to determine whether the data were normally distributed, and the mean values were compared between groups of common β-globin mutations using a t-test. Analysis was performed by SPSS v.22 for Windows, licensed for Universitas Padjadjaran.

## Results

**Fig. 2:**
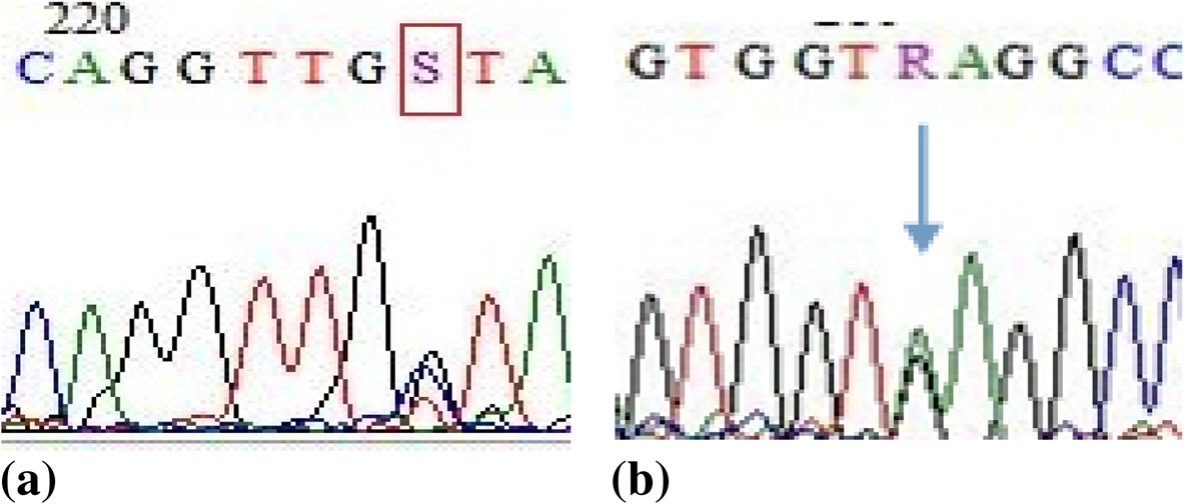
Sequences from individuals with mutations in the β-globin gene (HBB, NM_000518) at c92 + 5G > C (IVS1nt5) (**a**) or at c.79G > A (CD-26) (**b**)

Of 196 family members participating in a complete blood count examination, 117 had low MCV (< 80 fL) and/or low MCH (< 27 pg) and/or low Shine & Lal index (< 1530). The Hb electrophoresis results showed a high peak HbE level in 27 (13.7%) participants (mean 24.06% ± 0.95; range 22.4–26.5) (Table 1). Further sequencing analysis of the samples with high HbE levels revealed a mutation at codon (CD) 26 (c.79G > A) (Fig. 2b).

**Table 1:** Distribution of anaemia and Hb fraction analysis in association with β-globin gene mutations at c.79G > A (CD-26) and at c92 + 5G > C (IVS1nt5) among carrier thalassemia individuals from Bandung, Indonesia

	β-globin mutation						
	c.79G > A		(CD-26)	c92 + 5G > C		(IVS1nt5)	*P* value
Anaemic status	n	(%)		n	(%)		
Anaemic	7	(25.9)		16	(57.1)		0.019^#^
Not anaemic	20	(74.1)		12	(42.9)		
Hb fractions	Mean	± sd	(min-max)	Mean	± sd	(min-max)	
Hb E	24.06%	± 0.95	(22.4–26.5)	–			
Hb A2	3.18%	± 0.62	(2.6–3.8)^a^	4.65%	± 0.77	(4.0–5.6)	< 0.001^##^
Hb A	72.51%	± 0.18	(69.7–74.1)	95.23%	± 0.08	(94.4–96.2)	< 0.001^##^

Note: ^a^HbA2 is normal (2.2–3.2%) and borderline (3.3–3.9%)

Statistical significance is set at *p* < 0.05; ^#^χ-square test or ^##^t-test (data normally distributed)

The other samples with low MCV and/or low MCH and/or low Shine & Lal index and no HbE peak had various HbA2 levels, ranging from normal (n40), borderline (n20), and high (n30) levels of HbA2. *All* samples with high HbA2 levels (> 4%) had mutations at IVS1nt5 (c.92 + 5G > C; n28) (Fig. 2a), CD 8–9 (c27_28insG; n1) and CD 19 (c.59A > G; n1; known as HbMalay).

There was a significant difference (*p* < 0.001) in the HbA2 level between the mutation at CD26 (c.79G > A) and at IVS1nt5 (c.92 + 5 G > C), with mean values of 3.18% ±0.62 (range 2.6–3.8) and 4.65% ±0.77 (range 4.0–5.6), respectively (Table 1). Interestingly, anaemia was only found in 25 and 57% of the mutations at CD26 (c.79G > A) and at IVS1nt5 (c.92 + 5 G > C), respectively.

## Discussion

Thalassemia cases are becoming a global health problem due to population migration [11]. This disease is increasing in Indonesia and listed as the fifth in the national catastrophic disease, making thalassemia an emerging national burden. However, the screening of thalassemia carriers in Indonesia is not a mandatory yet. In some countries, screening of thalassemia carrier status is encouraged in basic health care services [11]. Moreover, cascade screening among family members has proven to be cost effective after identifying thalassemia carrier individuals [12]. Therefore, we have initiated screening of first degree family members of thalassemia patients for their carrier status.

Various formulas using a complete blood count have been developed to detect β-thalassemia carriers in areas where the prevalence of thalassemia major is high; however, specificity and sensitivity vary across regions [13]. Our study has shown that cell counter-based parameters such as the Shine & Lal index may serve as a good predictor to detect haemoglobinopathies at the earliest stage. Even though we only had eight parameters of complete blood count examination in our study, the Shine & Lal index < 1530 could predict *all* β-thalassemia carriers with mutations at c92 + 5G > C and c.79G > A. Both mutations are regarded as the most prevalent mutations among populations in West Java, Indonesia [10]. Interestingly, anaemia is only prevalent in 57.1 and 25.9% of β-thalassemia carrier IVS1nt5 (c92 + 5G > C) and HbE (c.79G > A) variants, respectively, indicating that most individuals have normal Hb or are non-anaemic, though they have low SLI. SLI has thus been proven to be a valid discriminating index to distinguish between IDA and β-thalassemia [14], as also shown in our previous study [15].

Furthermore, HbA2 analysis plays an important role in distinguishing IDA and β-thalassemia carriers [16], for example, anaemia with HbA2 > 4% indicates a β-thalassemia carrier with a mutation at IVS1nt5 (c92 + 5G > C). The increase in HbA2 becomes an important parameter for thalassemia carrier identification. However, samples with low SLI and normal or borderline HbA2 levels may suggest an IDA or possible α-thalassemia carrier with deletional or non-deletional α-globin gene defects, and this needs to be further analysed by molecular analysis [17]. The machine used in our study showed a high peak of HbE, which is clearly separated from HbA2 (Fig. 1a), and all confirmed a mutation at c.79G > A, while some machines showed a very high value of HbA2, indicating the HbE variant. Our study showed that the HbA2 fraction value for the HbE variant is normal or borderline, in concordance with the study by Mais et al. (2009) [18].

Mutations at IVS1nt5 (c.92G > C) and at CD 26 (c.79G > A) are common in Indonesia; however, both mutations might have different phenotypes [19]; therefore, molecular analysis is necessary. Since the DNA analysis is limited and expensive, screening strategies for thalassemia and HbE in rural communities in our region may be directed to previously published guidelines [20, 21]. Some researchers suggest a simple way to detect mutations using techniques such as the multiplex amplification refractory mutation system (ARMS) PCR or PCR-RFLP [22] rather than sequencing or the most recent diagnostic-based array [23].

This study had several limitations; the lack of family pedigrees to establish genotype/phenotype segregation is a major issue in this study. A better database of family members’ registries has recently been established. Another limitation of this study is that iron profile examination is lacking. Furthermore, respondents with a low Shine & Lal index who have normal or borderline HbA2 were not further examined for possible other α-globin gene mutations; therefore, DNA analysis must be performed since co-inheritance of IDA and β-thalassemia and α- and β-thalassemia might occur even in rare cases [24, 25].

## Conclusion

In a limited resource area such as Indonesia, the Shine & Lal index may have great value in predicting β-thalassemia carrier status in the general population. Wherever it is available, further screening of iron and HbA2 profiles is necessary; by all means, molecular examination is the most necessary examination to confirm the point mutation or deletion in α- or β-thalassemia carrier detection, which is needed to map the mutation spectrum among populations.

## Data Availability

Datasets used and/or analysed during the current study are available on reasonable request. Please email e.sahiratmadja@unpad.ac.id, cc. corresponding author amelani@yahoo.com.
